# Proximate Analysis and Techno-Functional Properties of *Berberis aristata* Root Powder: Implications for Food Industry Applications

**DOI:** 10.3390/foods13172802

**Published:** 2024-09-03

**Authors:** Ankita Awari, Mukul Kumar, Deepika Kaushik, Ryszard Amarowicz, Charalampos Proestos, Rizwan Wahab, Mohammad Rizwan Khan, Igor Tomasevic, Emel Oz, Fatih Oz

**Affiliations:** 1 Department of Food Technology and Nutrition, Lovely Professional University, Phagwara 144411, Punjab, India; awari118@gmail.com; 2 Department of Biotechnology, Faculty of Applied Sciences and Biotechnology, Shoolini University, Solan 173229, Himachal Pradesh, India; 3 Institute of Animal Reproduction and Food Research, Polish Academy of Sciences, 10-748 Olsztyn, Poland; 4 Laboratory of Food Chemistry, Department of Chemistry, School of Sciences, National and Kapodistrian University of Athens, 15772 Athens, Greece; harpro@chem.uoa.gr; 5 Zoology Department, College of Science, King Saud University, Riyadh 11451, Saudi Arabia; 6 Department of Chemistry, College of Science, King Saud University, Riyadh 11451, Saudi Arabia; 7 Faculty of Agriculture, University of Belgrade, Nemanjina 6, 11080 Belgrade, Serbia; 8 German Institute of Food Technologies (DIL), 49610 Quakenbrück, Germany; 9 Department of Food Engineering, Agriculture Faculty, Ataturk University, Erzurum 25240, Türkiyefatihoz@atauni.edu.tr (F.O.)

**Keywords:** characterization, glucose inhibition assay, *Berberis aristata* root, in vitro assay, phytochemical analysis, techno-functional properties

## Abstract

*Berberis aristata*, commonly known as Indian barberry, has been traditionally used for its medicinal properties. Despite its recognized pharmacological benefits, its potential application in the food industry remains underexplored. This study aims to investigate the proximate analysis and techno-functional properties of *Berberis aristata* root powder to evaluate its feasibility as a functional food ingredient. The root powder of *Berberis aristata* was subjected to proximate analysis to determine its moisture, ash, protein, fat, fiber, and carbohydrate content. Techno-functional properties, including water and oil absorption capacity, emulsifying and foaming properties, and bulk density, were evaluated using standardized analytical techniques. The proximate analysis revealed a high fiber content and a significant number of bioactive compounds. The root powder exhibited favorable water and oil absorption capacities, making it suitable for use as a thickening and stabilizing agent. Emulsifying and foaming properties were comparable to conventional food additives, indicating their potential in various food formulations. The findings suggest that *Berberis aristata* root powder possesses desirable techno-functional properties that could be leveraged in the food industry. Its high fiber content and bioactive compounds offer additional health benefits, making it a promising candidate for functional food applications. Further research on its incorporation into different food matrices and its sensory attributes is recommended to fully establish its utility.

## 1. Introduction

*Berberis aristata*, also known as daruharidra, daruhaldi, Indian barberry, tree turmeric, and Chitra, is a spiny, robust herb with a yellowish hue that belongs to the Berberidaceae family. It thrives in the sub-Himalayan region, the Nilgiri hills of southern India, and elevated areas of Nepal at altitudes ranging from 2000 to 3500 m [1]. Renowned for its medicinal properties, this plant holds significant importance in Ayurveda, Siddha, and Unani medicinal traditions [2]. Its roots, in particular, are valued for their therapeutic properties. *Berberis aristata* exhibits genetic variation across different regions of India. This variation can be influenced by factors such as geographic distribution, altitude, climate, and environmental conditions.

Traditionally, *Berberis aristata* has been utilized as a tonic, demulcent, diaphoretic, diuretic, and alternative remedy for various health conditions such as wound healing, skin disorders, rheumatism, snakebites, menorrhagia, jaundice, and ocular issues [3]. The principal alkaloid constituent found in various parts of the plant, including leaves, roots, rhizomes, and stem bark, is berberine. Early studies in 1988 demonstrated its hypoglycemic effects when used to manage diarrhea and diabetes [4]. In regions like India and Nepal, *Berberis aristata* is utilized in traditional medicine for alleviating allergies, metabolic disorders, cholera, acute diarrhea, latent malaria, amoebiasis, and ophthalmic concerns and as a natural laxative [5,6,7]. Berberine exhibits diverse pharmacological activities, serving as an antimicrobial, anti-inflammatory, analgesic, antipyretic, hepatoprotective, immunomodulatory, and even antidepressant agent [8].

A wide range of chemical constituents are present in various parts of *Berberis aristata*, with alkaloids being the primary components. The root bark, for instance, contains various protoberberine alkaloids such as Karachine, dihyrokarachine, tetrahydropalmatine, tetrahydroberberine, epiberberine, palmatine, palmatine dehydrocaroline, jatrorhizine, columbamine, and palmatine chloride [9]. Additionally, alkaloids like aromoline, oxyberberine, berbamine, oxyacanthine, and berberine chloride are extracted from the plant. Polyphenolic flavonoids such as quercetin, meratin, and rutin are found in the flower, along with E-caffeic acid and chlorogenic acid [10]. Other constituents include the aliphatic hydrocarbon n-docosane in the heartwood extract and heavy metals like cadmium (1.37 ppm), lead (8.70 ppm), chromium (1.07 ppm), zinc (56.5 ppm), and manganese in the rhizome [11,12].

*Berberis aristata* exhibits various pharmacological activities, including hepatoprotective effects, anti-inflammatory properties, anti-diabetic potential, anti-cancer activity, and inhibition of platelet aggregation and adhesion [2,13,14]. Its aqueous root extracts have been found to possess anti-inflammatory properties, while extracts from both *Berberis aristata* and *Coscinium fenestratum* demonstrate anti-inflammatory potential [2]. Berberine-containing extracts exhibit antibacterial and antimicrobial activities against various pathogens. Furthermore, ethanol and alcoholic stem extracts show anti-diabetic potential, and the plant’s DPP-IV inhibitory property suggests anti-diabetic efficacy [13].

The objective of this study was to analyze the proximate and techno-functional properties, along with the chemical characterization of *Berberis aristata* Linn root powder, to assess its potential health benefits and applications. Through a comprehensive examination of its composition and pharmacological activities, we aimed to understand its suitability for various pharmaceutical, nutraceutical, and functional food formulations. In particular, this study sought to explore its potential as a natural intervention for obesity and related metabolic disorders, thereby contributing valuable insights into the development of novel herbal-based treatments for weight management and overall human health.

## 2. Materials and Methods

### 2.1. Materials

#### Plant Material and Chemicals

*B. aristata* root samples were obtained from a local market in Kondwa, Pune, Maharastra, India, in February 2023. All the analytical-grade chemicals used in the experiments were sourced from the Department of Food Science and Technology at Lovely Professional University in Phagwara, Punjab, India.

### 2.2. Methods

#### 2.2.1. Herbal Powder Preparation

*B. aristata* roots were washed three times with tap water and once with distilled water to eliminate any foreign substances. Subsequently, the peels were dried in a tray drier (manufactured by B.S. Exports, Ambala, India) at a temperature of 50 °C ± 2.0 °C for 24 h. Once completely dry, the peels were finely powdered using a grinder (USHA Rapidmix 500-Watt, Gurugram, India) and then stored in a ziplock bag at room temperature [15].

#### 2.2.2. Determination of Total Yield of *B. aristata* Root Powder

To assess the yield, we employed a customized sieving technique inspired by the procedure outlined by Manuwa et al. [16]. The sample underwent sieving in a shaker set to 60 amplitude for 20 min using a test sieve with a mesh size of 250 µm. Following sieving, the weight of the sample was recorded, and the yield was determined by dividing the weight of the sieved powder (in grams) by the initial weight of the sample (in grams), then multiplying by 100.

#### 2.2.3. Proximate Analyses

The moisture content evaluation of the samples adhered to the methodology delineated by Haryanto et al. [17], ash analysis was performed according to the procedure outlined by Bharadwaj et al. [18], crude fat analysis was executed following the approach detailed by Mohd Daud Khassetia [19], protein analysis was conducted based on the method described by Sharma and Dhuria [20], and fiber analysis was carried out as per the method by Akter et al. [21].

#### 2.2.4. Tapped Density and Bulk Density

The determination of bulk density (ρbulk) entails assessing the total volume occupied by both solid particles and void spaces within a divided powder. The calculation of bulk density for *B. aristata* root powder followed the procedure delineated by Etti et al. [22]. In this method, the powder was poured into a 50 mL measuring cylinder up to the 50 mL mark. Then, the cylinder was dropped from a height of 1 inch onto a firm wooden surface at 2-s intervals, and the resultant volume of the powder was meticulously measured.

Tap density (ρtap) presents an alternate measure of bulk density, achieved by tapping or vibrating the container in a specific manner. This tapping or vibration encourages a more orderly arrangement of particles, leading to increased particle packing density. Determining tap density involves recording the initial volume or mass of the powder. Subsequently, the measuring cylinder or vessel containing the powder undergoes mechanical tapping for 500 repetitions. Periodic measurements of volume or mass are recorded until minimal or no further changes are observed. Tapped density serves as an indicator of the powder’s compactness after mechanical tapping. Consequently, tap density typically surpasses bulk density.

#### 2.2.5. Hausner’s Ratio and Carr’s Index

The Carr Index and Hausner Ratio are measures used to assess the flow properties of a powder, as outlined by Mochahary et al. [23]. The Carr Index (CI) is calculated by dividing the difference between the tapped density and bulk density by the tapped density. According to Carr’s flowability index, powders with a Carr Index ranging from 5% to 15% are classified as having excellent flowability, while a Carr Index exceeding 25% typically indicates poor flowability. On the other hand, the Hausner Ratio (HR) is a parameter utilized to evaluate powder flow characteristics. It is computed by dividing the tapped density by the bulk density. HR values falling within the range of 1.0 to 1.1 indicate easy-flowing powder, while HR values between 1.1 and 1.25 suggest moderate flowability. An HR exceeding 1.25, up to 1.4, indicates poor flow, and an HR surpassing 1.4 signifies a powder with extremely low flowability.

#### 2.2.6. Angle of Repose (°)

The procedure for determining the angle of repose (θ) for *B. aristata* powder followed the methodology described in the study conducted by Dattatraya et al. [24]. A specific quantity of the dried powder was placed into a funnel positioned 6 cm above a level surface. The powder was allowed to flow freely, forming a mound on a sheet of paper placed horizontally. Throughout this process, precise measurements of the height and radius of the powder heap were taken and recorded. The angle of repose serves as a crucial indicator for assessing the flow characteristics of the powder. Typically, an angle of repose around 30 degrees signifies smooth flow, indicating good flowability. Conversely, angles of repose approaching 40 degrees usually suggest sluggish flow, indicating diminished flowability.

#### 2.2.7. Scanning Electron Microscopy (SEM)

The *B. aristata* root powder underwent morphological characterization utilizing scanning electron microscopy (SEM) employing a JEOL microscope. The samples were scrutinized at different magnifications, including 100×, 200×, 500×, 1000×, and 2000×, following the methodology outlined by Kumar et al. [25].

#### 2.2.8. Plant Extract Preparation

A modified extraction method, as detailed by Kumar et al. [26], was utilized to procure acetone extracts. To obtain these extracts from the herbal materials, 1 g of powdered herb was dissolved in 10 mL of ethanol at a temperature of 35 °C. Subsequently, this solution was placed in an orbital shaker and incubated at 15.75 relative centrifugal force (RCF) for 48 h. Following the incubation period, the tubes underwent centrifugation at 35,203.58 RCF for 5 min. Subsequent filtration was conducted using Wattman Filter Paper 1, and the extracts were stored at a refrigerated temperature of 3 °C to allow solvent evaporation and drying for 8 days. The weight of each extract was measured to determine the yield as a percentage. The final extract was stored in sealed glass vials at −18 °C for further analysis.

#### 2.2.9. Total Phenolic Content (TPC) Determination

The determination of total phenolic content (TPC) in the ethanol extracts was conducted with specific adaptations based on the methodology outlined by Alara et al. [27]. In a test tube, a solution comprising 300 μL of the ethanol extract was combined with 1 mL of ethanol and 200 μL of Folin–Ciocalteu reagent. Following a 5 min incubation, 600 μL of a 0.02 mM sodium carbonate solution was added, followed by a 2 h incubation at 40 °C. To establish calibration, a blank solution was prepared using ethanol in lieu of the extract, and a gallic acid standard curve was generated. The absorbance of the resulting solutions was measured at 765 nm using a UV-spectrophotometer (Visible Spectrophotometer 168, Ahmedabad, India). The outcome was expressed as milligrams of gallic acid equivalents per gram of dried weight sample (mg GAE/g d.w.).

#### 2.2.10. Total Flavonoid Content (TFC) Estimation

The quantification of total flavonoid content (TFC) in the ethanol extracts followed the protocol developed by Alara et al. [27]. *B. aristata* powder was dissolved in ethanol at a concentration of 500 μg/mL. In a test tube, 300 μL of the extract was mixed with 3.4 mL of a 30% aqueous methanol solution. Subsequently, solutions of sodium nitrite (0.5 M) and aluminum chloride (0.3 M) were added. After a 5 min incubation, sodium hydroxide solution (1 M) was introduced, and the mixture was thoroughly mixed before measuring the absorbance at 506 nm against a blank using a Visible Spectrophotometer 168. Results were expressed as μg of Quercetin equivalents (QE) per mg of extract.

#### 2.2.11. DPPH Estimation

DPPH radicals were generated by dissolving about 0.004 g of DPPH reagent in 100 mL of ethanol. For the assay, 20 μL of the extract was mixed with 180 μL of DPPH. The mixture was kept in the dark for 15 min, and absorbance at 517 nm was measured using a Visible Spectrophotometer 168. The analysis included blank samples with only ethanol and standard samples with ascorbic acid in ethanol. The extracts were assessed at various concentrations to determine the IC_50_, representing the concentration causing a 50% reduction in DPPH absorbance [27,28].

#### 2.2.12. Amylase Inhibition Assay

The assessment of amylase inhibitory potential followed the protocol outlined by Kumar et al. [26]. A substrate solution was prepared by dissolving 500 mg of soluble starch in 25 mL of 0.4 M NaOH, heating it at 100 °C for 5 min, adjusting the pH to 7.0 with HCl, and diluting it to 100 mL with distilled water. Various concentrations of plant extract solutions were prepared using acetate buffer (pH 6.5). In a microplate well, 20 μL of the sample and 40 μL of the substrate solution were mixed, followed by the addition of 20 μL of α-amylase solution (50 μg/mL). After a 15 min incubation at 25 °C, the enzymatic reaction was halted by adding 80 μL of 0.1 M HCl, followed by 200 μL of 1 mM iodine solution. The optical density was then measured at 650 nm using a visible spectrophotometer (Visible Spectrophotometer 168).

#### 2.2.13. Glucose Uptake Assays Using Dialysis Bag

The glucose uptake assay was conducted following the method described by Kumar et al. [26]. A dialysis membrane with a pore size of 2000 KDa (6 cm × 15 mm) was used. The membrane was pre-soaked in distilled water to aid expansion. One end of the dialysis tube was sealed, and 1 mg of herbal extract was placed inside along with 10 mL of the dialysis solution and glucose was used as a control in this assay. Subsequently, 15 mL of a 0.22 mM glucose solution was added and thoroughly mixed with the herbal extract. The system was then incubated at 37 °C for 4 h. After incubation, centrifugation was performed at 35,203.58 RCF for 20 min. Another centrifuge tube containing 45 mL of 0.15 M NaCl was prepared. Glucose movement from the dialysis membrane to the external solution was monitored at different time intervals using a glucometer (Control D Glucometer, Delhi, India).

#### 2.2.14. Fourier Transform Infrared Spectroscopy (FTIR)

In accordance with the methodology described in the literature by Asema et al. [28], Fourier Transform Infrared Spectroscopy (FTIR) analysis was conducted. Each sample, approximately 5 mg, was blended with 5 mg of KBr to facilitate qualitative assessment. Subsequently, the dehydrated and powdered state of each sample was examined using a PERKIN ELMER FTIR spectrometer equipped with a KBr beam splitter. The spectra obtained were analyzed following the guidelines provided by Stuart. The FTIR spectrophotometer operated at a resolution of 0.5 cm^−1^, utilizing Spectrum 10 software. The spectral range analyzed spanned values from 400 cm^−1^ to 4000 cm^−1^.

#### 2.2.15. High-Performance Liquid Chromatography (HPLC)

HPLC system employed in this analysis was equipped with a CBM 20 elite controller, DGU-20 A5 prominence online degasser, LC-20AD binary pumps, SIL-20A prominence auto sampler, CTO-20 prominence column oven, and SPD-20A prominence UV-Vis detector, all from Shimadzu Co., Kyoto, Japan. Chromatographic separation was conducted using a reversed-phase C_18_ analytical column with dimensions of 4.6 × 150 mm and a particle size of 5 µm. The mobile phase consisted of 0.1% TFA (A) and Acetonitrile (B) (in a proportion of 60:40, *v*/*v*). The system maintained a constant flow rate of 1 mL/min throughout the run, and the column temperature was 30 °C. For both standards and samples, 10 µL was injected into the system, and the berberine peak was monitored at 350 nm. The total run time was set to 25 min, following a methodology inspired by previous research conducted by Shigwan et al. [29].

#### 2.2.16. Gas Chromatography/Mass Spectrophotometry

A Shimadzu 6890 GC/MS system equipped with an HP-INNOWax column (60 m × 0.25 mm × 0.25 m; Agilent Co., Haryana, India) was utilized. The sample was introduced using split injection with a ratio of 10:1 and a 2 mm direct injector liner, along with a 15 m Alltech EC-5 column (250 μ I.D., 0.25 μ film thickness). The oven temperature program began at 35 °C, held for 2 min, and then ramped at 20 °C per minute to 450 °C, where it was maintained for 5 min. Helium was used as the carrier gas at a flow rate of 2 mL/min. Flavor compound identification was achieved by comparing their spectra to a library (APAWLY-9781119376743 NIST Mass Spectral Library 2017). The relative percentage composition of each component was determined by comparing its average peak area to the total areas. Mass spectra and chromatograms were managed using the GCMSsolution™ software. This methodology was adapted from Sharmila et al. [30].

#### 2.2.17. Statistical Analysis

The study conducted a set of experiments in triplicate and presented the results as the mean value along with the standard deviation. To assess significant differences among the means, they employed a statistical method known as one-way analysis of variance (ANOVA), followed by the Duncan post hoc test, with a significance level set at *p* < 0.05 [16].

## 3. Results

### 3.1. Proximate Analysis

The proximate analysis of *B. aristata* root is enlisted in Table 1. The moisture content of the sample was found to be 6.10%, which aligns closely with the results reported by Paudel et al. [31] at 6.82%. The fiber content was determined to be 43.34%, a value similar to the 43.84% documented in *B. lyceum* by Shah et al. [32]. The protein content of 4.69% falls within the range reported in Ahmad’, 2009’s study on *B. lyceum* Royal root, which documented values between 4.4% and 6.24%. Furthermore, the ash content, measured at 2.86%, corresponds to the value reported in Srivastava et al. [33] examination of *Berberis asiatica*.

### 3.2. Techno-Functional Properties

The techno-functional properties of daruhaldi powder were assessed to understand its behavior and suitability for various applications as shown in Table 2. The powder exhibited a tap density of 0.65 ± 0.05 gm/cm^3^ and a bulk density of 0.61 ± 0.13 gm/cm^3^, indicating its mass per unit volume under tapped and untapped conditions, respectively. With a Hausner’s ratio of 1.28 ± 0.21, daruhaldi powder demonstrated moderate flowability, which is essential for processes such as blending and tableting in pharmaceutical manufacturing. The angle of repose, measuring the stability of the powder pile, was found to be 21.32 ± 0.43 degrees, suggesting good flow properties, facilitating handling and processing. Carr’s ratio, reflecting compressibility, was calculated to be 5.22 ± 0.34, indicating moderate compressibility, which is advantageous for achieving uniformity in dosage forms. Overall, these results indicate that daruhaldi powder possesses consistent techno-functional properties, which are crucial for its utilization in pharmaceutical and manufacturing applications, ensuring desirable flow and compressibility characteristics that contribute to efficient processing and high-quality end products.

### 3.3. Phytochemical Analysis

The Total Phenolic Content (TPC) and Total Flavonoid Content (TFC) of daruhaldi (turmeric) samples were investigated, revealing noteworthy concentrations of phenolic and flavonoid compounds as shown in Table 3. The TPC of 11.42 ± 0.92 mg gallic acid equivalents per gram of sample (mg GAE/g) suggests a substantial presence of phenolic compounds, comparable to 111.8 µg of gallic acid equivalent in the aqueous extract by Sharmila et al. [30], and 11.04 ± 2.20 μg/mg of GAE in the ethanol extract by Bhatnagar et al. [34]. Similarly, the TFC of 2.33 ± 1.03 mg quercetin equivalents per gram of sample (mg QE/g) indicates a notable content of flavonoids, consistent with a total flavonoid content of 6.08 ± 0.50 μg/mg of QE in the aqueous extract and 288.3 µg of quercetin equivalent in the ethanol extract. Moreover, the DPPH assay demonstrated the antioxidant potential of daruhaldi, with the sample exhibiting activity 20 µg/mL less than that of *B. vulgaris*—Skrad origin root, according to Končić et al. [35]. These findings collectively highlight daruhaldi’s rich phenolic and flavonoid compositions and its promising antioxidant properties, suggesting potential health benefits associated with its consumption.

### 3.4. Scanning Electron Microscopy (SEM)

**Figure 1:**
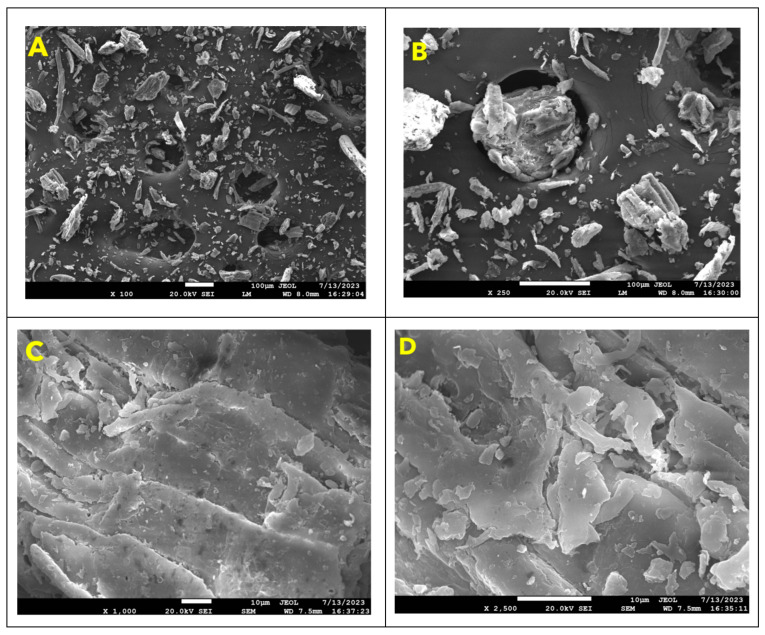
Scanning electron microscopy (SEM)for *B. aristata* powder at different magnifications. (**A**) ×100 magnification (**B**) ×250 magnification (**C**) ×1000 magnification and (**D**) ×2500 magnification.

In this investigation, we present a Scanning Electron Microscope (SEM) image (Figure 1) showcasing the intricate details of mineral formations. Captured at 250× magnification with an accelerating voltage of 20.0 kV in secondary electron imaging (SEI) mode, the image unveils a diverse assortment of particles characterized by varying shapes, surface textures, and sizes. While some particles appear elongated, others exhibit irregular angular structures.

The roughness observed in the SEM images of daruhaldi powder can be ascribed to several factors, encompassing the botanical origin of the material, processing methodologies, and inherent structural properties. Daruhaldi roots are renowned for harboring various bioactive compounds, including alkaloids and flavonoids, which could contribute to the heterogeneous surface morphology observed. Additionally, factors such as drying techniques, particle size distribution, and environmental conditions during processing may influence the surface texture of herbal powders.

From a practical perspective, the rough surface morphology of daruhaldi powder holds implications for its functional properties and practical applications. The heightened surface area conferred by the rough texture has the potential to augment interactions with solvents or biological matrices, thereby impacting its dissolution kinetics, bioavailability, and therapeutic effectiveness [36]. Moreover, the roughness may exert an influence on the flowability, dispersibility, and rheological characteristics of daruhaldi powder across various formulations.

### 3.5. Fourier Transform Infrared (FTIR)

**Figure 2:**
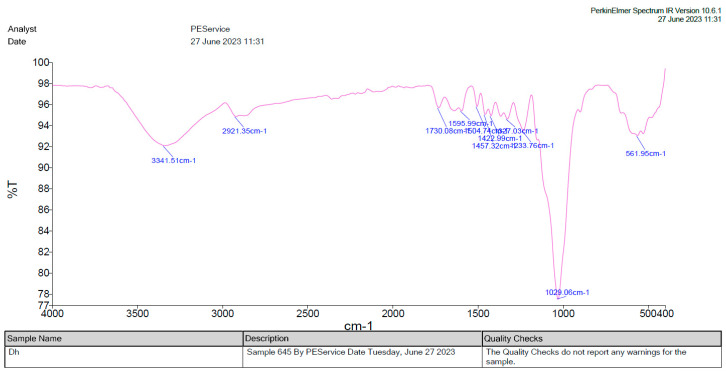
Fourier transform infrared (FT-IR) for *B. aristate* root extract.

Figure 2 depicts Fourier-transform infrared (FTIR) analysis of daruhaldi, revealing 11 peaks, suggesting the presence of a complex molecule. In the single bond region (2500–4000 cm^−1^), a broad absorption band between 3650 and 3250 cm^−1^ confirmed the existence of hydrate (H2O), hydroxyl (-OH), ammonium, or amino groups. A peak at 3341.51 cm^−1^ was identified as NH stretching of a primary amine, as reported by Gupta et al. [37] in their study on the crystal properties of celecoxib. Additionally, a narrow band at 2921.35 cm^−1^ indicated aliphatic compounds.

In the double bond region (1500–2000 cm^−1^), three peaks were observed. One peak at 1730.08 cm^−1^, corresponding to C=O stretching vibration, suggested the presence of simple carbonyl compounds such as ketones, aldehydes, esters, or carboxyl. This peak also indicated the formation of an ester bond between two galloyl groups, confirming the presence of hydrolyzable tannins, as discussed by Shtewi et al. [38] in their research on *Mentha piperita* leaf extract. Another peak at 1595.99 cm^−1^ was assigned to conjugated carbonyl or aromatic ring stretching vibrations.

In the fingerprint region, five peaks were identified: 1457.32 cm^−1^, 1422.99 cm^−1^, 1327.03 cm^−1^, 1233.76 cm^−1^, and 1029.06 cm^−1^. The peak at 1422.99 cm^−1^ was attributed to symmetric stretching vibrations of the carboxyl group by Pal and Banat [39], while the peak at 1327.03 cm^−1^ was specified to aromatic amines by Mathai et al. [40]. The remaining peaks in this region were not explicitly assigned in the provided data.

### 3.6. High Performance Liquid Chromatography (HPLC)

**Figure 3:**
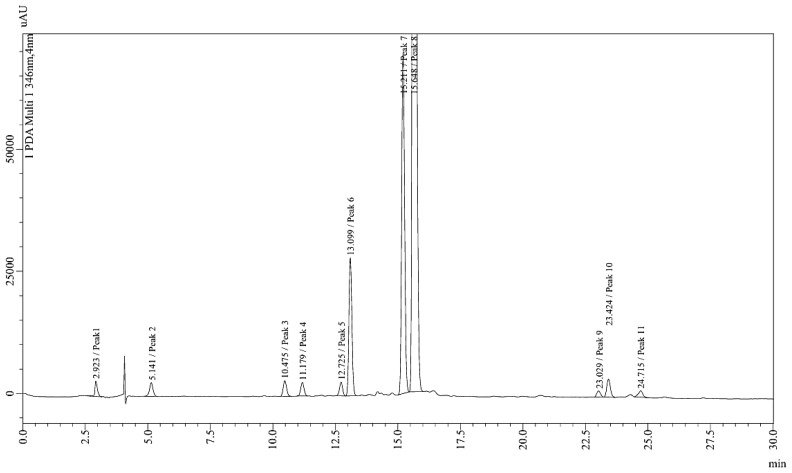
High-performance liquid chromatography (HPLC) for *B. aristate* root extract.

A total of 11 peaks were observed in the chromatogram depicted in Figure 3. The identification of berberine occurred at a retention time of 5.141 min, representing approximately 0.621% of the total peak area. Notably, this peak closely resembled the berberine peak reported by Das et al. [41], which was observed at a retention time of 5.011 min. The detailed data of the chromatogram are as follows: Peak 1 had a retention time of 2.923 min with an area of 21435, constituting 0.497% of the total area. Peak 2 (berberine) appeared at 5.141 min with an area of 26784, accounting for 0.621%. Peak 3 had a retention time of 10.475 min and an area of 29905, representing 0.693%. Peak 4 was observed at 11.179 min with an area of 25,459, making up 0.590%. Peak 5 had a retention time of 12.725 min with an area of 24,230, contributing 0.562%. Peak 6 appeared at 13.099 min with an area of 233,467, constituting 5.412%. Peak 7 had a retention time of 15.211 min and an area of 566,073, representing 13.123%. Peak 8 was observed at 15.648 min with an area of 3,320,403, making up 76.975%. Peak 9 had a retention time of 23.029 min with an area of 13,150, contributing 0.305%. Peak 10 appeared at 23.424 min with an area of 36,901, accounting for 0.855%. Lastly, Peak 11 was observed at 24.715 min with an area of 15,828, representing 0.367% of the total area. The sum of all peak areas amounted to 4,313,636, equating to 100% of the total peak area.

### 3.7. Gas Chromatography-Mass Spectrophotometry (GC-MS)

**Figure 4:**
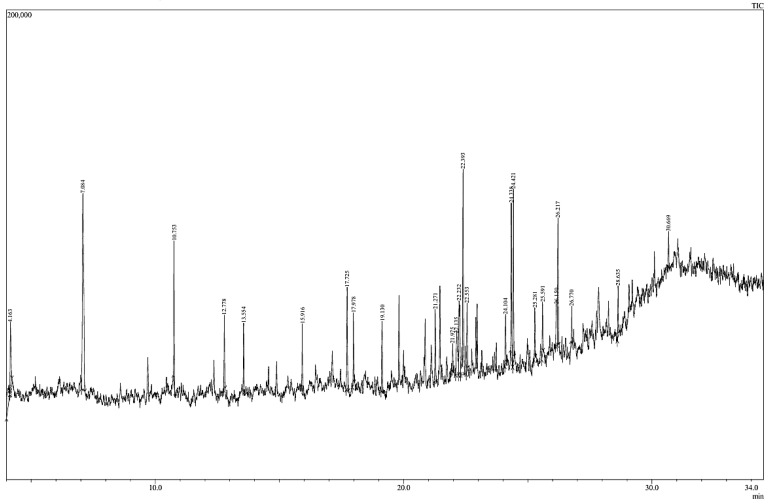
Gas Chromatography Mass Spectrophotometry (GCMS) for *B. aristata* root extract.

The exploration of active constituents within *B. aristata* root powder encompassed an analysis of the GC-MS chromatogram, as shown in Figure 4. This investigation, performed on the ethanol extract of *B. aristata* root powder, revealed the presence of a total of 26 distinct compounds at different retention time, peak area, and functional group, such as Amphetamine (4.020, 2.62) Alcohol derivative; Glycerin (4.163, 3.58) Ester; 3-Methylbenzyl alcohol, TBDMS derivative (7.084, 16.52) Silane; Methyl 6,6,8,8,10,10-hexamethyl-3-oxo-2,5,7,9,11-pentaoxa-6,8,10-trisilatridecan-13-oate (10.753, 4.66) Ether; Silane, cyclohexyldimethoxymethyl- (12.778, 2.52) Acid derivative; 2,2′-Bis-trimethylsilylbenzhydryl methyl ether (13.554, 2.15) Phenol derivative; 2,5-Dihydroxybenzoic acid, 3TMS derivative (15.916, 2.13) Ether; 2,4-Di-tert-butylphenol (17.725, 4.26) Ester; Mollugin, trimethylsilyl ether (17.978, 2.46) Alkane; Bis[2-(3,5-di-tert-butylbenzoyloxy)-1-naphthyl]methane (19.130, 3.40) Amine; Butane, 1,4-bis(9,10-dihydro-9-methylanthracen-10-yl)- (21.271, 2.78) Oxazolidinone; 1-tert-Butyl-1,1-dimethyl-N-(4-nitrobenzyl)silanamine (21.975, 0.97) Amine; 2-Oxazolidinone, 3-amino-5-(4-morpholinylmethyl)- (22.135, 3.61) Amine; 1-tert-Butyl-1,1-dimethyl-N-(4-nitrobenzyl)silanamine (22.232, 5.61) Fatty acid derivative; Hexadecanoic acid, methyl ester (22.393, 9.81) Acetylacetonate; Aluminum tris(acetylacetonate) (22.553, 2.57) Fatty acid derivative; 7-Hexadecenoic acid, methyl ester, (Z)- (24.104, 1.76) Fatty acid derivative; Methyl stearate (24.338, 5.87) Organosulfur compound;1-Isobutylsulfanylmethyl-2,8,9-trioxa-5-aza-1-sila-bicyclo[3.3.3]undecane (24.421, 6.23) Amine; Dipropylamine, N-(3-butenyl)- (25.281, 1.80) Silane; 1-Cyclohexyldimethylsilyloxy-3,5-dimethylbenzene (25.591, 1.97) Alkane; Tetracosane (26.150, 3.33) Silane; 1-Cyclohexyldimethylsilyloxy-3,5-dimethylbenzene (26.217, 5.22) Phosphine; Ethyne, bis(dicyclohexylphosphino)- (26.770, 1.07) Phenol; Phenol, 4,4′-methylenebis[2,6-bis(1,1-dimethylethyl)- (28.635, 1.88) Alcohol derivative; 2,2,4-Trimethyl-3-(3,8,12,16-tetramethyl-heptadeca-3,7,11,15-tetraenyl)-cyclohexanol (30.669, 1.23) Phenol.

### 3.8. α-Amylase Inhibition Assay

The data from the α-amylase inhibition assay highlight the potential of daruhaldi (turmeric) to inhibit α-amylase activity, an important enzyme involved in carbohydrate breakdown, with implications for managing weight and addressing obesity-related issues. Table 4 outlines the inhibition percentages across different concentrations of daruhaldi, ranging from 20 μg/mL to 100 μg/mL, chosen deliberately to explore its dose-dependent effects on α-amylase inhibition. Additionally, the table includes the IC_50_ value, indicating the concentration required to inhibit 50% of α-amylase activity.

The results demonstrate significant α-amylase inhibitory activity of daruhaldi, with inhibition percentages increasing as concentrations rise. Particularly noteworthy is the IC_50_ value of 22.43 μg/mL, indicating effective suppression of amylase activity even at lower concentrations through non-competitive inhibition. In a similar study by Bhatnagar et al. [34], comparable results were reported when analyzing Molecular Dynamics (MD) simulations of berbamine, alloxanthine, protopine, and benazepril, along with the reference molecule acarbose, to assess the stability of the docked protein-ligand complex of the daruharidra plant. These screened ligands exhibited highly negative binding energies upon interaction with the α-amylase enzyme, suggesting their potential efficacy in inhibiting α-amylase activity.

These findings suggest that daruhaldi holds promise in impeding carbohydrate digestion and absorption by targeting α-amylase function, which could have implications for regulating postprandial glucose levels, crucial for weight management, and combating obesity-related metabolic disorders. This inhibition assay can also be useful in controlling diabetes as By inhibiting α-amylase, the breakdown of complex carbohydrates into simpler sugars is slowed down, leading to a gradual release of glucose into the bloodstream. This can help prevent sudden spikes in blood glucose levels after meals, a critical aspect of managing diabetes. Additionally, controlling postprandial glucose levels can contribute to better glycemic control over time, reducing the risk of diabetes-related complications such as cardiovascular diseases, neuropathy, and nephropathy. Therefore, compounds or natural products with significant α-amylase inhibition activity, such as daruhaldi (turmeric), hold promise as adjunctive therapies in the management of diabetes mellitus.

### 3.9. Glucose Inhibition Assay

**Figure 5:**
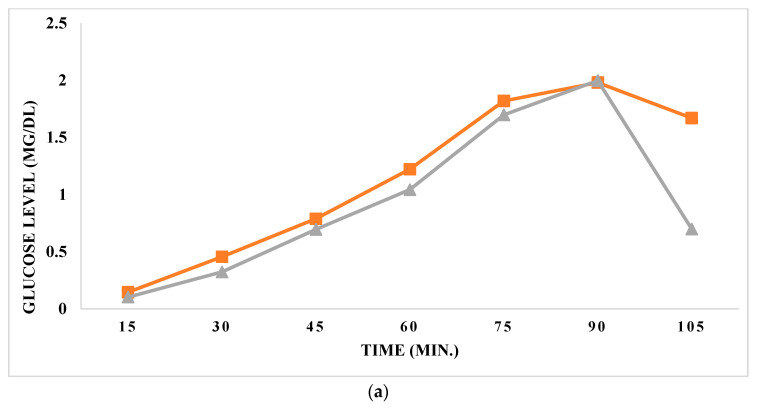

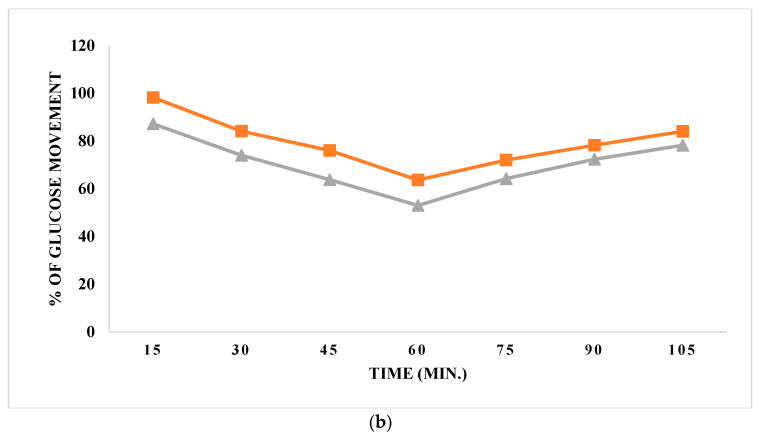
(**a**) Represents the glucose level (mg/dL), where the orange line represents the control, and the grey line denotes *B. aristate* root extract. (**b**) Represents the glucose movement percentage (%), where the orange line represents the control, and the grey line denotes *B. aristate* root extract.

The glucose concentrations measured in milligrams per deciliter (mg/dL) across various time points (in minutes) during an investigation involving daruhaldi as shown in Figure 5. Additionally, it provides the IC_50_ value, indicating the concentration necessary to inhibit 50% of a specific activity. Glucose levels initiate at 0.356 mg/dL at 15 min, gradually escalating over time. The determined IC_50_ value for daruhaldi is 1.86, underscoring its effectiveness in inhibiting the specified activity. Various studies have tied the effectiveness of daruhaldi with obesity work by Kim et al. [42] shows that berberine treatment enhances AMPK activity in adipocytes, resulting in elevated GLUT1 expression and improved glucose uptake. This mechanism can mitigate insulin resistance, a characteristic feature of obesity, by optimizing glucose utilization and reducing blood glucose levels. Ultimately, the actions of berberine may offer therapeutic advantages for addressing obesity and associated metabolic disorders.

Compounds that impede glucose uptake could potentially curb excessive glucose absorption by adipocytes, thereby restricting fat accumulation and supporting weight management endeavors. By intervening in glucose uptake pathways, such compounds may present therapeutic opportunities for individuals grappling with obesity and its related metabolic complications.

## 4. Discussion

The study aimed to explore the proximate analysis and techno-functional properties of *Berberis aristata* root powder to evaluate its feasibility as a functional food ingredient. The proximate analysis demonstrated that the root powder is rich in fiber and bioactive compounds. The high fiber content aligns with previous research that highlights the nutritional benefits of dietary fibers in promoting digestive health and reducing the risk of chronic diseases (e.g., cardiovascular disease, type 2 diabetes). The significant presence of bioactive compounds further supports its potential health benefits, given the known antioxidant, anti-inflammatory, and antimicrobial properties of *Berberis aristata*. The techno-functional properties analysis revealed favorable water and oil absorption capacities, suggesting that *Berberis aristata* root powder can function effectively as a thickening and stabilizing agent in food products [43]. This property is particularly valuable in the formulation of sauces, dressings, and baked goods, where maintaining consistency and texture is crucial. The emulsifying and foaming properties were also comparable to those of conventional food additives, indicating that this root powder can serve as a natural alternative to synthetic emulsifiers and foaming agents in various food applications. The results of this study are consistent with previous findings on the nutritional and functional properties of berberis species. For example, studies on *Berberis vulgaris* have also reported high fiber content and beneficial techno-functional properties, supporting the potential of this genus as a source of functional ingredients in the food industry. However, this study is among the first to comprehensively analyze *Berberis aristata* root powder, filling a critical gap in the literature and expanding the potential applications of this underutilized plant. The findings suggest several practical applications for *Berberis aristata* root powder in the food industry. Its high water and oil absorption capacities make it suitable for use in moisture-retention and fat-replacement applications, which are valuable in low-fat and gluten-free product formulations. The emulsifying and foaming properties could be leveraged in the production of dairy and non-dairy beverages, desserts, and bakery products [44,45,46,47,48,49]. Additionally, the presence of bioactive compounds offers an added health benefit, which can be marketed to health-conscious consumers seeking functional foods with natural ingredients. While the study provides valuable insights into the properties of *Berberis aristata* root powder, there are limitations that should be addressed in future research. Firstly, the sensory attributes of food products containing this root powder were not evaluated. Sensory analysis, including taste, texture, and overall acceptability, is crucial for determining consumer acceptance. Secondly, the bioavailability and stability of the bioactive compounds during food processing and storage need to be investigated to ensure the retention of health benefits in the final product.

## 5. Conclusions

The study aimed to analyze the proximate composition, techno-functional properties, phytochemical content, and pharmacological activities of *Berberis aristata* Linn root powder. Proximate analysis revealed moisture content (6.10%), fiber content (43.34%), protein content (4.69%), and ash content (2.86%), closely aligned with previous studies. Techno-functional properties indicated moderate flowability and compressibility, crucial for pharmaceutical processes. Phytochemical analysis showed significant Total Phenolic Content (TPC) (11.42 ± 0.92 mg GAE/g) and Total Flavonoid Content (TFC) (2.33 ± 1.03 mg QE/g), with promising antioxidant activity. HPLC analysis identified berberine as a significant component of Berberis aristata, constituting approximately 0.621% of the total peak area. The α-amylase inhibition assay revealed that daruhaldi effectively inhibits carbohydrate digestion, with an IC_50_ value of 22.43 μg/mL, indicating its potential in managing obesity. Furthermore, glucose inhibition assay results demonstrated daruhaldi’s efficacy in reducing excessive glucose absorption. These findings collectively highlight the potential of *Berberis aristata* Linn root powder as a natural intervention for obesity and related metabolic disorders, aligning with the study’s objective to assess its suitability for pharmaceutical, nutraceutical, and functional food applications.

## Figures and Tables

**Table 1:** Proximate analysis for *B. aristata* powder.

Proximate Analysis Parameters	Percentage Value (%)
Moisture content	6.10 ± 0.34
Ash content	2.86 ± 0.17
Fibre content	43.34 ± 0.26
Fat content	2.67 ± 0.02
Protein estimation	4.69 ± 0.67

Data are presented as mean ± SD (*n* = 3)

**Table 2:** Techno functional properties for *B. aristata* root powder.

Techno Functional Properties	Values
Tap Density (gm/cm^−3^)	0.65 ± 0.05
Bulk Density (gm/cm^−3^)	0.61 ± 0.13
Hausner’s ratio	1.28 ± 0.21
Angle of repose (°)	21.32 ± 0.43
Carr’s ratio	5.22 ± 0.34

Data are presented as mean ± SD (*n* = 3).

**Table 3:** Phytochemical analysis for *B. aristata* powder.

Parameters	Values
Total phenolic content (GAE/g)	11.42 ± 0.92
Total flavonoid content (QE/g)	2.33 ± 0.53
DPPH (% inhibition)	20 ± 0.94

Data are presented as mean ± SD (*n* = 3).

**Table 4:** α-amylase inhibition assay for *B. aristata* powder.

Inhibition Assay	Concentration (μg/mL)	% of Inhibition	IC_50_
α-amylase inhibition assay	20	40.37	22.43
	40	77.56	
	60	80.43	
	80	85.65	
	100	96.77	

## Data Availability

The original contributions presented in the study are included in the article, further inquiries can be directed to the corresponding authors.
